# Investigation of Significant Parameters during Abrasive Waterjet Turning

**DOI:** 10.3390/ma14164389

**Published:** 2021-08-05

**Authors:** Adam Štefek, Jan Raška, Libor M. Hlaváč, Sławomir Spadło

**Affiliations:** 1 Department of Physics, Faculty of Electrical Engineering and Computer Science, VSB-Technical University of Ostrava, 17. listopadu 2172/15, 70800 Ostrava-Poruba, Czech Republic; libor.hlavac@vsb.cz; 2 PWR Composite s.r.o., Sadová 1892/41, 70200 Ostrava-Moravská Ostrava, Czech Republic; jan@pwr.cz; 3 Department of Materials Science and Materials Technology, Faculty of Mechatronics and Mechanical Engineering, Kielce University of Technology, al. Tysiąclecia Państwa Polskiego 7, 25314 Kielce, Poland; sspadlo@tu.kielce.pl

**Keywords:** abrasive water jet, turning, impact angle, traverse speed, rotational speed

## Abstract

This paper presents an investigation of abrasive waterjet turning (AWJT). The purpose of the article was to investigate significant parameters of the turning process and to evaluate their impact on the turning product. The influence of the traverse speed, the rotational speed, and the relative position of the jet to the specimen (lateral jet shift) were investigated. Based on the previous research done in this field, the multi-pass tangential turning method was selected. Rotational speed does not seem to have a significant impact on the AWJ turning process. However, the relative position of the jet is a key parameter for improving the efficiency of the process. Increasing the lateral jet shift causes the volume of the material removed to increase until the optimal impact angle is reached. These findings need to be extended in order to adjust AWJT. Without these improvements, a comparison of jet to traditional technologies is inappropriate.

## 1. Introduction

Abrasive waterjet (AWJ) cutting is a widely used method in industry because it can cut almost every type of material. It has been developed continuously since the 1980s and today is even able to perform 3D machining. The development of jet cutting enables applications in various industries. For example, it is increasingly used in the exploitation of mineral resources [1]. This success has principally been based on analytical physical models and a huge amount of experimental work. However, AWJ can also be used for unconventional applications. One of these is a process whereby a jet erodes a rotating specimen to a certain diameter and shape (abrasive waterjet turning (AWJT)). When examining AWJT, we can benefit from the knowledge and models used for abrasive water jet cutting. The traverse speed, pressure, stand-off distance, abrasive flow rate, and angle of impact are the most useful variables for controlling and modifying the AWJ properties during the cutting process. During the turning process, we must account for several new variables influencing the performance of the jet. These are associated with the rotational movement of the specimen and with the relative position of the focusing tube. The specimen can rotate either clockwise or counter-clockwise, with a different frequency. Therefore, the direction of motion and the rotational speed/frequency are the new variables.

**Figure 1:**
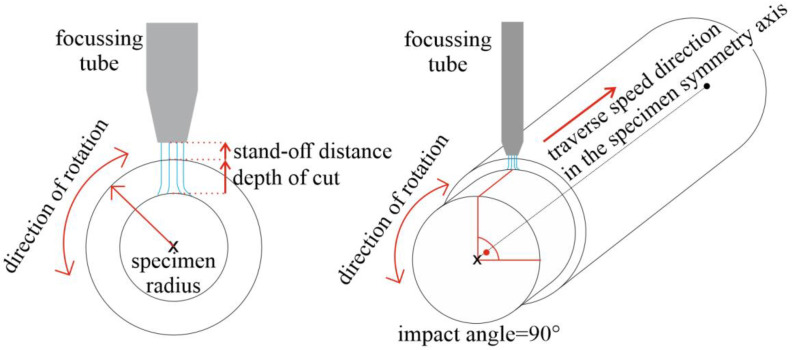
Scheme of the radial AWJT mode.

**Figure 2:**
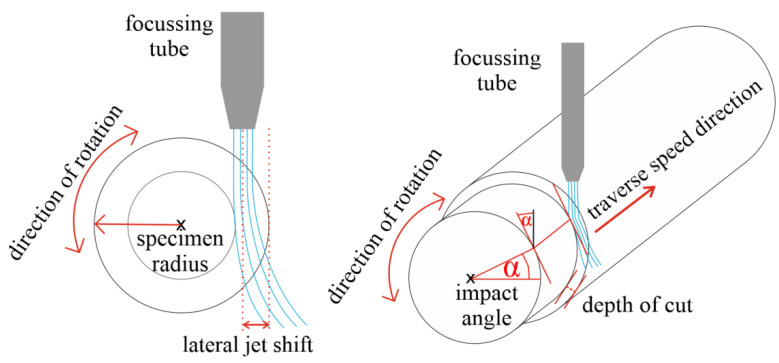
Scheme of the tangential AWJT mode.

In terms of the relative position of the tube to the surface, we can distinguish the radial mode and the tangential mode of AWJT. If we put the focusing tube right above the symmetrical axis of a specimen and move the jet only along this axis, we speak about radial mode turning. In case we do not the tilt focusing tube, the impact angle is 90°, so this mode is very similar to AWJ cutting or drilling (Figure 1). If the focusing tube is placed arbitrarily above the surface and the impact angle is varied (in extreme cases, tangential to the surface), we speak about the tangential (or offset) mode (Figure 2). In this case, the lateral jet shift is a new variable influencing the performance of the jet.

Hashish investigated the influence of some parameter changes on material removal rate and waviness when using AWJT [2]. The traverse speed and the pressure were chosen as the main parameters affecting and quality of the process, compared to the rotational speed, which was considered a negligible parameter. Ansari et al. tried to visualize the whole process from two perspectives [3]. Similar to AWJ cutting, the step formation and jet deflection were noticeable during the experiments. They observed that the material removal rate of aluminum 6061-T651 increased with the increase of the lateral jet shift and the traverse speed, until the limit values were reached [4]. However, this deteriorated the quality of the surface. The precision of AWJT technology was also studied by Hashish [5].

Zhong and Han observed that increasing the rotational speed improved the final surface of a turned glass specimen [6]. Hashish suggested that to obtain the required diameter of a specimen with a high material removal rate, several passes of the jet have to be made with a certain traverse speed [7]. Hlaváč and Palička studied the influence of certain AWJ parameters on precision with various materials [8]. Axinte et al. mentioned AWJ turning as an option for profiling and dressing grinding wheels [9]. The most profitable strategy is to use lateral jet shifts significantly higher than the jet diameter, to increase the material removal rate. In order to create a smoother surface, a final improving pass with a lower lateral jet shift should be performed. Several other researchers investigated the influence of AWJ parameters on the material removal rate, the final diameter, and the quality of the surface, using both studied modes. They studied high performance materials [10], turned alumina ceramics [11], tried to prepare sandstone samples [12], and machined carbon-fiber-reinforced plastics [13], wood plastic composites [14], and aluminum alloys [15].

The influence of the process variables has been studied since the beginning of AWJ studies, as is evident from publications [2,5,8,15,16,17]. The influence of abrasive materials has been studied [18], as well as the material removal rate and the final surface roughness, with a changing traverse speed and abrasive flow rate on plastic materials [19].

Models of the AWJT process date back to the 1990s. Bouzid et al. derived both numerical and experimental models of AWJ orthogonal turning [20]. A parameter prediction model was prepared by Zeng et al. [21]. Manu and Babu created an analytical model based on the erosion model of the tangential mode and the physical equations determining the final diameter of the specimen [22]. Another model based on physical equations and the finite element method was proposed [23].

Weiyi et al. made a comparison of the tangential and the radial mode [24]. The main problem with using of the radial mode seems to be the inability of predicting and controlling the depth of cut, and the poor quality of the resulting surface. To obtain a better surface, the tangential (offset) mode can be used for the final cut. The radial mode models were made to predict the depth of cut and the roughness of the surface [24,25].

Although these articles provide very useful information, it is difficult to exploit these models in new research. Statistical models may only be valid for certain measuring conditions, and there are always several variables which are hard to obtain. Analytical models, which are mostly based on physical laws and assumptions, can be used to a greater extent. However, there is still a problem in determining the values of some variables and even about the assumptions that were made to determine or calculate them.

This paper is focused on a better understanding of the tangential mode of the AWJT process. Its aim was to find the essential parameters and to evaluate the whole process of the tangential mode of AWJT.

## 2. Theoretical Background

Compared to AWJ cutting, several new variables were defined. They are related to the relative position of the jet and specimen and to the rotational movement. These new variables are the direction of rotation, the rotational speed, the lateral jet shift, and the radius of the specimen. The theoretical influence of the new variables, as well as some already well examined variables, will be discussed below.

*Direction of Rotation*

We can divide the direction of rotation according to the action of the jet. Research has mostly focused on the direction parallel to the impinging jet, because this should provide a better final surface quality. However, it is also assumed that if the jet acts against the movement of the specimen, a higher material removal rate could be reached.

*Traverse Speed and Rotational Speed*

The traverse speed is mostly used to change the AWJ properties and, therefore, the performance of the jet. It defines the duration of AWJ cutting. However, during AWJ turning, the time of machining is also affected by the rotational speed. Therefore, the variable “number of passes” (***N***), which counts with both motions, was defined:

(1) $$N=f\frac{D}{v_{p}}$$

Since the traverse speed determines how many particles will hit the surface of the specimen, it plays a dominant role in the performance. The influence of rotational speed was tested.

*Lateral Jet Shift*

**Figure 3:**
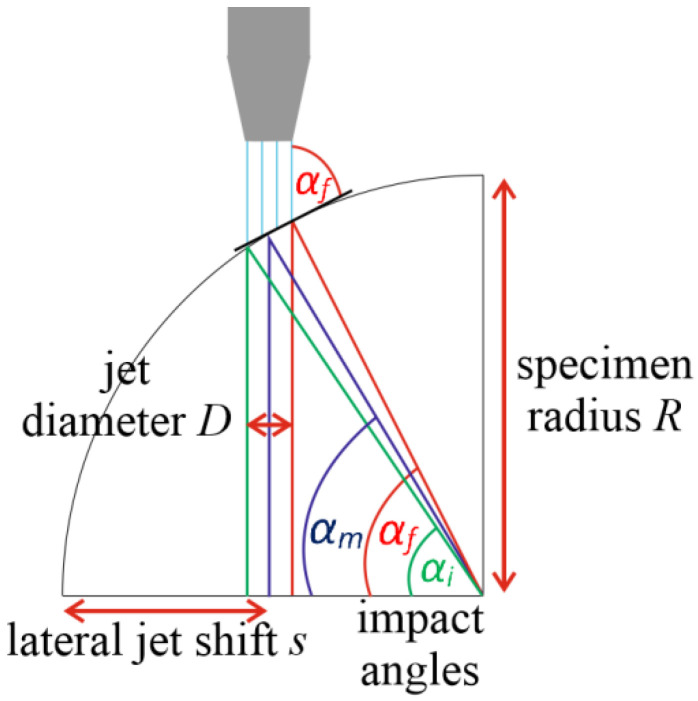
Relationship between lateral jet shift and impact angle.

This is a variable which is relevant only for tangential AWJ turning. The lateral jet shift (***s***) is a pre-set machine length of the feed into the material (Figure 3). In the case of using the multi-pass tangential AWJT mode, the number of the lateral shifts into the material (***N**_**s**_*) is also set. Several new variables, which are related to the lateral jet shift, were defined. Their main purpose is to interpret the AWJT multi-pass results appropriately.

The distance which is counted as the sum of the machine made lateral shifts into the material is called the cumulative lateral jet shift (***s**_**c**_*).

To obtain information about the actual AWJT performance and the relative position of the jet with respect to the variable diameter of the samples, the lateral jet position inside the specimen diameter (***JP***) was defined. This variable indicates the radial distance between the edge of the sample and the axis of the center of the focusing tube. This is measured from the cumulative lateral jet shift and the actual diameter/radius of the specimen. It reflects the volume removal rate during every pass.

As the jet itself has a diameter, a single angle of impact is not defined, but a set of them. This angle of impact also depends on the diameter of the specimen. The bigger the sample diameter, the lower the set of impact angles for a selected shift. We can define three impact angles based on the diameter of the specimen and the relative position of the focusing tube (Figure 3).

(2) $$\alpha_{i}=\arccos\left(\frac{R-s+\frac{D}{2}}{R}\right)$$

(3) $$\alpha_{m}=\arccos\left(\frac{R-s}{R}\right)$$

(4) $$\alpha_{f}=\arccos\left(\frac{R-s-\frac{D}{2}}{R}\right)$$

$\alpha_{i}$—the initial angle of impact,$\alpha_{m}$—the medium angle of impact, and $\alpha_{f}$—the final angle of impact. The initial and the final angle represent the endpoints of the set of impact angles.

## 3. Experimental Setup

Three different experiments were performed. In the first experiment, the influence of the rotational frequency and the traverse speed were investigated on the round and squared samples. The second one focused on the changing respective lateral jet shift on the round samples. The last experiment was conducted to evaluate the quality of the final surface.

The experiments were performed in the Laboratory of Liquid Jet at the VŠB-Technical University of Ostrava. An abrasive waterjet cutting table PTV WJ1020-1Z-EKO (PTV s.r.o., Hostivice, Czech Republic) and pump HSQ 5X (Flow Int., Seattle, WA, USA) were used, and the invariables setup is summarized in Table 1.

**Figure 4:**
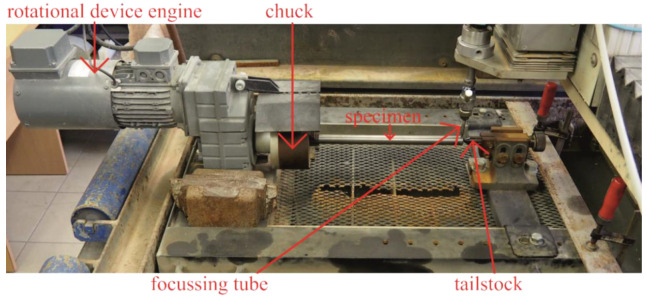
The experimental setup with parts of the rotational device highlighted.

A special device was developed and constructed to provide rotational movement of the specimen. This device works independently from the abrasive waterjet machine and is able to a reach rotational speed up to 1000 rpm (Figure 4).

Since these experiments served as a new investigation of the process, the common 1.0038 steel grade was used (Table 2).

All the round and square rods were made of 1.0038 steel. The round samples were 20 mm in diameter and the samples with a square cross-section had an side of 18 mm.

**Figure 5:**
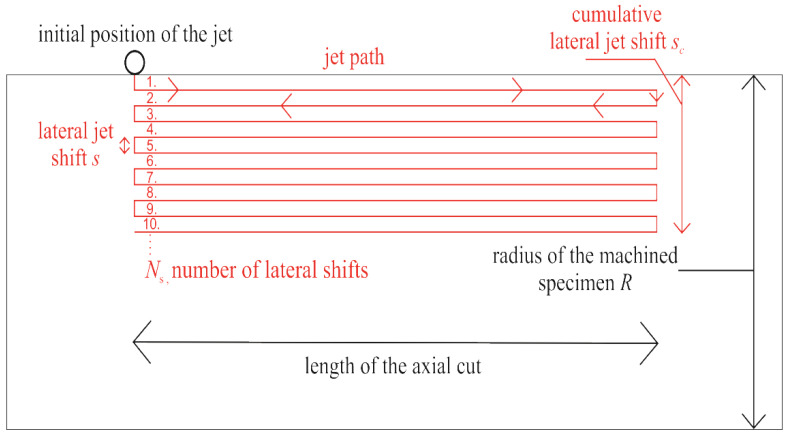
Top view of the jet trajectory during AWJ turning of the rounded specimen.

The AWJT tangential mode with multiple passes was chosen for each of the experiments. In order to simplify the whole process, only simple straight passes of the jet were produced (Figure 5).

After every experiment, the final diameter of the specimen was measured with a digital caliper (0.01 mm precision). Each diameter was measured at least ten times and these values were then averaged. Furthermore, the standard deviation of the measured diameter values was calculated and multiplied by two to increase the accuracy. This value was considered the resulting measurement uncertainty.

Alicona Infinite Focus was used to measure the roughness, specifically the largest height of the profile Rz and the mean arithmetic deviation of the profile Ra. WENZEL LH 65 X3M PREMIUM(WENZEL Group GmbH Co. KG, Wiesthal, Germany) was used to measure the roundness of the final sample. Eleven points on the samples were measured. From these measured values and the device coordination system, a figure was created. The Gaussian method was used to process measured data and determine the estimated circle.

The method is based on determination of the center circle, in which the sum of the squares of the deviations from the specified profile is the smallest. The output value of the measurement is the plotted body of the “circle” and the average value of the deviation from the center circle.

## 4. Results and Discussion

*Investigation of the Influence of the Traverse Speed and Rotational Frequency on the Volume Removed*

The experiment was focused on monitoring the effect of the traverse speed and the rotational frequency on the volume of material removed. Several combinations of both of these variables were chosen (Table 3). The remaining unchanged parameters are listed in Table 1.

The experimental traverse speed set was defined based on experience with steel machining. The speed value of 10 mm/min should guarantee an ideal machining of the sample to the required diameter. On the contrary, a value of 50 mm/min should ensure insufficient removal. The remaining speeds were chosen between these extreme values. Three rotational frequencies were chosen based on the limits of the engine, and 1000 RPM was the maximal frequency it was able to reach. The rest were chosen as fractions of the maximal value.

For each rotational frequency, all the specified traverse speeds were tested (a total of 12 tests). In the first part the round rods were machined.

A higher number of feeds into the material should reduce the effect of possible inaccuracies due to inaccurate initial jet position settings. Another reason for choosing a higher number for the lateral displacements into the material is that the effects associated with the change of the tested quantities will be more pronounced. It was assumed that the low lateral jet shifts should also provide an accurate final diameter.

Nine shifts into the material were chosen, with a single shift 0.5 mm long, so that the final diameter was 11 mm. Before each experiment, the focusing tube was positioned above the middle axis of the specimen, with the stand-off distance of 2 mm. Then the focusing tube was displaced in the radial direction to the edge of the sample, so that it “touches” the sample tangentially and only with an edge part of the jet. The length of the cut was 2 cm. The calculated number of passes is shown in Table 4, and the final diameters of the specimen are presented in Table 5.

**Figure 6:**
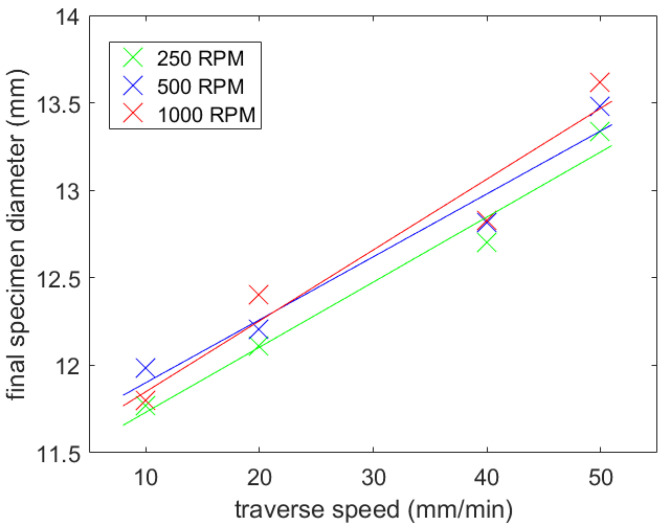
Influence of the traverse speed and the rotational speed on the final diameter (round rod).

It is easily visible from the results that the traverse speed still played a dominant role in volume of material removed. Increasing the traverse speed decreased the volume removal ratio, due to the shorter exposure time of the material to the jet (Figure 6).

**Figure 7:**
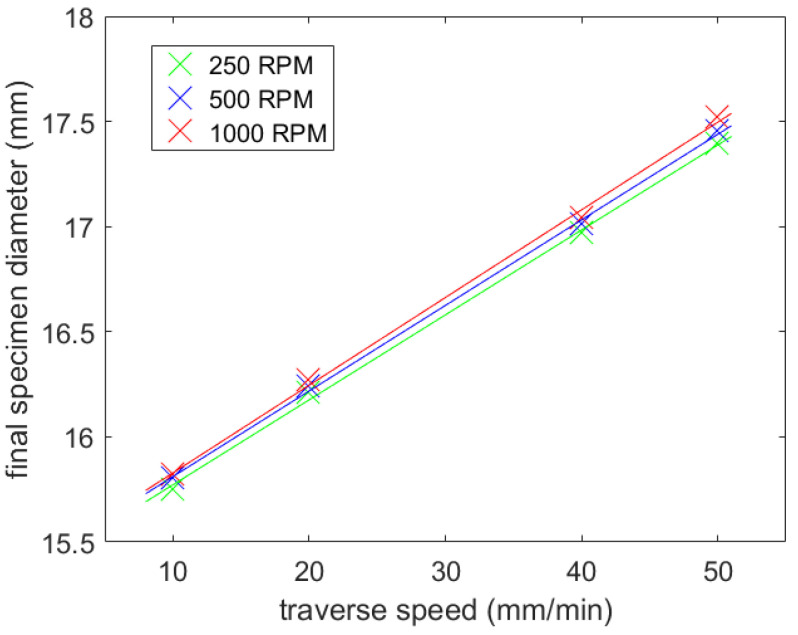
Influence of the traverse speed and the rotational speed on the final diameter (square rod).

The second experiment was performed on the square rods, with the results shown in Figure 7. The same parameters were used as in the experiment with the round rod (Table 3), except for the number of the lateral jet shift increments. Ten lateral jet shift increments were used, each with the width of 0.5 mm. This setup should ensure a final circular cross-section of the sample.

It can only be estimated whether increasing rotational speed also increases the resulting sample diameter, and thus lowers the volume of the material removed. This phenomenon is partially visible in both figures: Figure 6 and Figure 7. However, it is obvious that the impact of the rotational speed of the specimen on the material volume removed was low. This may have been caused by the type of material selected. It is generally harder to observe slightly changed conditions in high strength materials, such as steel. Since several lateral shifts were performed, we still lacked information about how much material was eroded during each jet shift.

*Investigation of Volume Removed for Different Lateral Jet Shifts*

To obtain more precise information about how much material was removed during each pass/lateral shift into the material, another experiment was performed. In this experiment, ten consecutive cuts were made into the round samples. The first cut was performed with just one lateral shift into the material, the second was performed with two lateral shifts, and this process continued until all ten shifts were reached. After that, the diameter of each cut was measured and compared with the previous one. Three different lateral jet shifts were selected and the medium traverse speed and rotational frequency were used (Table 6 and Table 7).

The number of shifts for each lateral jet shift is displayed in Table 7. Before each cut, the jet was positioned tangentially to the specimen surface in such a position that it did not touch the sample.

Each cut was compared with the previous one. Several new variables were created and computed to evaluate the turning process:

- Initial, medium, and final angle of impact $\alpha_{i}$, $\alpha_{m}$, $\alpha_{f}$ (Figure 3) and the range of the impact angles for each lateral shift.
- Change of the specimen radius $\Delta R=R_{i+1}-R_{i}$ (***i*** represents the number of the lateral jet shift) for each lateral shift.
- Volume of material removed for each lateral shift $V=D\left(\pi R_{i+1}^{2}-\pi R_{i}^{2}\right)$.
- Lateral jet position inside the specimen diameter (***JP***), since we were unable to turn the specimen ideally (the jet position did not determine the final radius of the specimen). This was calculated from the cumulative lateral jet shift and the actual diameter after a new shift.

Results of these measurements are shown in Table 8, Table 9 and Table 10.

**Figure 8:**
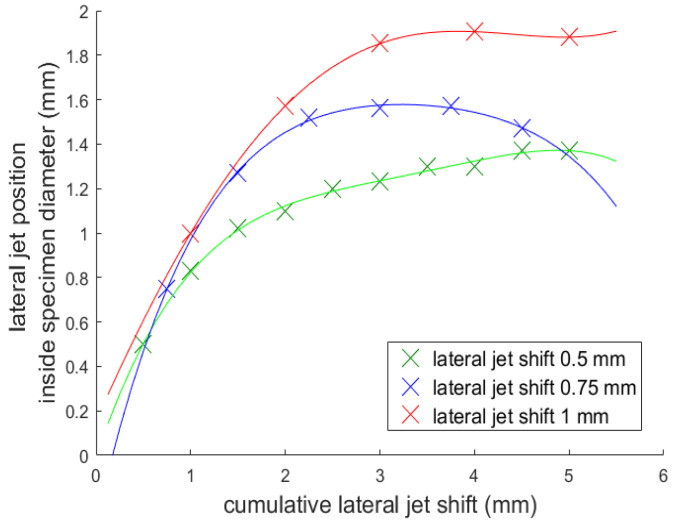
Cumulative lateral jet shift; comparison of the lateral position inside the specimen diameter.

**Figure 9:**
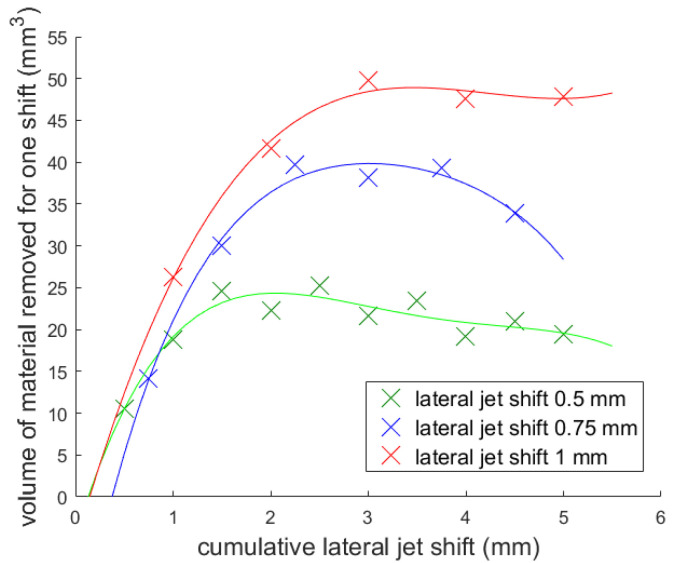
Dependence of the material removed on the jet cumulative lateral jet shift.

The dependence of ***JP*** on the cumulative lateral jet shifts into the material and the dependence of the volume of the material removed on the lateral jet shifts are shown in Figure 8 and Figure 9.

It is visible from the tables that the radius of the specimen was not only determined by the jet position. Figure 8 shows that the jet did not manage to remove a sufficient amount of material so that the lateral jet position inside the specimen diameter was constantly increasing. It is also visible, that the set of impact angles was large and, therefore, a greater original diameter of the specimen would have been more suitable.

Another problem is that the volume of material which we are able to remove decreases with the increasing of the cumulative lateral jet shift. This is clearly visible in Figure 9 and is caused by the round shape of the specimen. This trend was most visible for the 0.5 mm lateral jet shift. It seems that with this setup, the material removal was almost ideal compared to the other lateral jet shifts.

The material removal for the lateral shifts 0.75 mm and 1 mm was not sufficient (the volume removed did not, by some distance, reach the theoretically removable volume). Therefore, the results with these shifts are more suitable for discussion of the influence of angle of impact. From these results, an angle of impact around 32° seemed to correlate with the highest volume removed. However, more experiments need to be performed to confirm this.

*Investigation of the Final Shape and Quality of the Cut on Different Sample Shapes*

To evaluate the final surface quality, the roughness and roundness of the round samples were measured. The average roughness of the profile (***R**_**a**_*) and the mean peak to valley height of the roughness profile (***R**_**z**_*) were measured (Table 11 and Table 12).

The quality of the surface deteriorated with increasing traverse speed/decreasing exposure time (Table 11). Therefore, less material was removed and each pressure fluctuation was more visible on the sample as a striation.

The rotational frequency also seems to have an impact on the surface quality. The best surface quality was measured at 500 rpm in two cases. This may indicate that the frequency of rotation affects the contact time of the abrasive jet and the surface of the material. However, the scale of this phenomenon was so small that it could not be seen from the previous less precise measurements. In addition, we do not have a sufficient number of results, so more investigations need to be made for proper conclusions about this factor.

**Figure 10:**
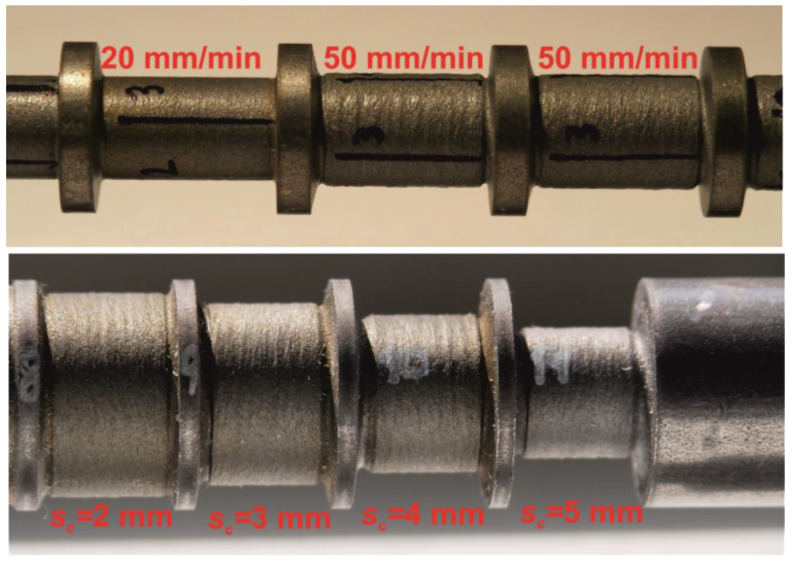
Comparison of the surface quality for two traverse speeds and several cumulative lateral jet shifts.

An apparent increase of the surface roughness was also related to an increase of the cumulative lateral jet shift (Table 12). A similar explanation for this phenomenon may be used. As said before, the energy of the AWJ was not sufficient to ideally turn the specimen. Owing to this, after each lateral shift (machine lateral feed) into the material, the ***JL*** increased. Therefore, there was more material to turn after each shift, and each fluctuation of the pressure was more visible through the striations (Figure 10).

A different phenomenon was visible for the lateral jet shift of 1 mm, where the last value of the roughness decreased. This may have been caused by more effective material removal during the higher cumulative lateral jet shift.

**Figure 11:**
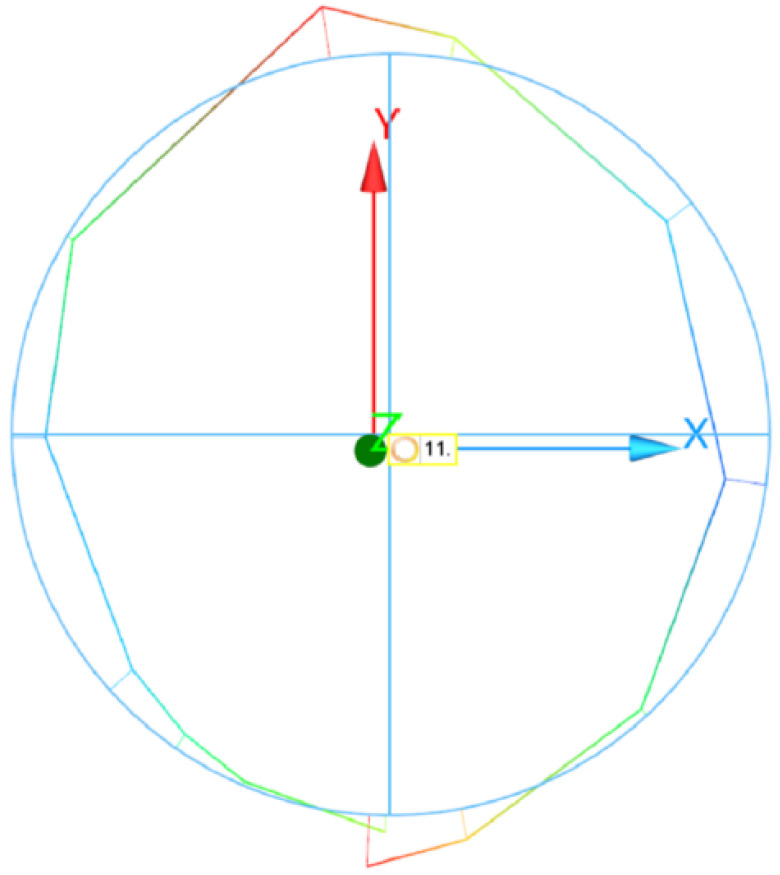
Visualization of measured points, and comparison to the circular shape.

The roundness of the samples was also measured. However, the results seemed to be rather random, and no relevant information was found. Therefore, only a picture of the final surface shape is presented here. The final sample shapes, measured on the individual parts of the machined rod, were similar in all experiments (Figure 11).

The origin of this shape may have been in the wrong alignment of the sample. Some distractions may also have been caused by the striations on the sample surface.

## 5. Conclusions

The aim of this research was to evaluate some parameters of the AWJT process and, at the same time, to better understand the material removal itself. Therefore, several experiments were performed to test the effect of the progression speed and the rotational frequency. Changes in the process speed were expected during the turning operation. The effect of the rotational frequency was proven to be low, in line with the previous findings of other researchers, but it may be more significant on other, easily machinable materials. In addition, the effect of the relative position of the abrasive waterjet and the rotating sample was tested. In almost all experiments the material removal was not ideal, and the jet position itself did not guarantee that the desired sample diameter was obtained. However, the experiments enabled determining at approximately which angle of impact the highest material removal occurred. The influence of the studied parameters on the turning process of special materials, such as composites, will be further investigated, using knowledge from this research.

Several results of the experiments are highlighted below and will be used in subsequent research:

- The traverse speed plays a dominant role in the volume removed compared to the rotational speed. An increasing traverse speed lowers the volume of material removed. The rotational speed does not seem to play a significant role in AWJT machining. According to the given results, it is assumed that with increasing rotation frequency the amount of removed material slightly decreases. To deeper evaluate the influence of the rotational speed, an easier to machine material should be selected.
- Although the traverse speed was set very low (10 mm/min), as well as the lateral jet shift (0.5 mm) and the rotational frequency (250 rpm), the AWJ had difficulty reaching the desired diameter. However, this may have been caused by an inaccurate setup of the initial jet position. Another reason for this observation may have been the low number of lateral jet shifts. Due to this setup, only the edge parts of the jet, with a lower energy, removed material during the final cut. The setting of the initial position of the jet should be improved to clearly determine the origin of this inaccuracy.
- The higher lateral jet shift during multi-pass AWJT is related to the higher volume removal rate. However, it was also connected with the worse final quality of the surface. Therefore, the best strategy is to perform the cut with a high lateral jet shift, and only the final cut should be made with a low lateral jet position, inside the specimen diameter, to improve the surface quality and guarantee the desired diameter.
- The highest roughness, 22.521 μm, was measured for a traverse speed 50 mm/min and 1000 RPM, and the lowest roughness, 5.65 μm, was measured for 10 mm/min and 250 RPM. These measurements contributed to the previous mentioned assumptions.
- The most suitable angle of impact for material removal rate seems to be around the value 32° for 1.0038 steel. The lateral shift, which reflects this impact angle, should be measured and used to increase the efficiency of the process. However, a small diameter specimen was used in these experiments and, therefore, a large set of impact angles occurred during the cutting. Specimens with a large diameter should be used in future research.
- The theoretical volume of the specimen to be removed depends on the lateral jet shift for circular specimens. For a small lateral jet shift (0.5 mm), the jet has enough time to machine a specimen sufficiently. Therefore, a decrease of the volume removed with an increasing total lateral jet shift is apparent. This explanation is also supported by the high quality of the surface with a small lateral jet shift.

## Figures and Tables

**Table 1:** Setup of basic experimental variables of the abrasive waterjet (the fixed variables).

Experimental pressure (MPa)	380
Water orifice diameter (mm)	0.25
Focusing tube diameter (mm)	1.02
Focusing tube length (mm)	76
Abrasive mass flow rate (g/min)	250
Abrasive material average grain size	80 MESH
Type of abrasive	Australian garnet

**Table 2:** Mechanical properties of the used steels.

Steel grade (WNR norm)	1.0038
Tensile strength ***σ**_**m**_* (MPa)	400
Yield strength ***σ**_**y**_* (MPa)	260
Young’s modulus ***E*** (GPa)	207
Brinell scale ***HB***	130

**Table 3:** Experimental setup with different combinations of traverse speed and rotational frequency.

Lateral jet shift ***s*** (mm)	0.5
Number of lateral jet shifts ***N**_**s**_*	9
Traverse speed ***v**_**p**_* (mm/min)	10, 20, 40, 50
Rotational frequency ***f*** (rpm)	250, 500, 1000

**Table 4:** Number of passes ***N*** for each setup.

Rotational Frequency (rpm)	Traverse Speed (mm/min)			
	10	20	40	50
250	25	12.5	6.25	5
500	50	25	12.5	10
1000	100	50	25	20

**Table 5:** Final diameters of the specimens (mm).

Rotational Frequency (rpm)	Traverse Speed (mm/min)			
	10	20	40	50
250	11.77 ± 0.02	12.19 ± 0.02	12.70 ± 0.01	13.33 ± 0.02
500	11.98 ± 0.01	12.20 ± 0.01	12.81 ± 0.01	13.48 ± 0.02
1000	11.80 ± 0.01	12.40 ± 0.02	12.82 ± 0.01	13.61 ± 0.02

**Table 6:** Experimental setup with different lateral jet shifts.

Length of the cut (mm)	12
Traverse speed (mm/min)	20
Rotational frequency (rpm)	500

**Table 7:** Number of lateral jet shifts.

Lateral Jet Shift (mm)	Number of Lateral Jet Shifts
0.5	10
0.75	6
1	5

**Table 8:** Experimental results with the lateral jet shift 0.5 mm.

Number of Lateral Jet Shifts	1	2	3	4	5	6	7	8	9	10
cumulative lateral jet shift (mm)	0.5	1	1.5	2	2.5	3	3.5	4	4.5	5
diameter after the respective jet shift (mm)	19.66 ± 0.03	19.05 ± 0.02	18.2 ± 0.02	17.41 ± 0.02	16.46 ± 0.02	15.6 ± 0.02	14.61 ± 0.02	13.75 ± 0.02	12.74 ± 0.01	11.73 ± 0.02
change of the specimen radius (mm)	0.17	0.31	0.42	0.4	0.47	0.43	0.5	0.43	0.5	0.51
lateral jet position inside the specimen diameter ***JP*** (mm)	0.5	0.83	1.02	1.1	1.2	1.23	1.3	1.3	1.37	1.37
volume removed V (mm^3^)	10.5	18.76	24.63	22.32	25.21	21.68	23.48	19.11	20.99	19.47
medium impact angle (°)	13	20	23	25	27	28	30	31	33	34
range of impact angles (°)	0–18	14–24	19–27	21–28.5	23–30.5	24–32	26–33.5	27–35	29–37	30–38

**Table 9:** Experimental results with the lateral jet shift 0.75 mm.

Number of Lateral Jet Shifts	1	2	3	4	5	6
cumulative lateral jet shift (mm)	0.75	1.5	2.25	3	3.75	4.5
diameter after respective jet shift (mm)	19.54 ± 0.01	18.54 ± 0.01	17.12 ± 0.01	15.64 ± 0.02	13.95 ± 0.02	12.3 ± 0.02
change of the specimen radius (mm)	0.23	0.5	0.71	0.74	0.85	0.82
lateral jet position inside the specimen diameter ***JP*** (mm)	0.75	1.272	1.52	1.56	1.57	1.47
volume removed V (mm^3^)	14.16	30.03	39.72	38.16	39.34	33.87
medium impact angle (°)	16	25	29	31	32	33
range of impact angles (°)	0–22	19–30	23.5–33	25–35	26.5–37	26–38

**Table 10:** Experimental results with the lateral jet shift 1 mm.

Number of Lateral Jet Shifts	1	2	3	4	5
cumulative lateral jet shift (mm)	1	2	3	4	5
diameter after respective jet shift (mm)	19.14 ± 0.01	17.71 ± 0.02	15.81 ± 0.03	13.76 ± 0.03	11.34 ± 0.02
change of the specimen radius (mm)	0.43	0.72	0.95	1.03	1.21
lateral jet position inside specimen diameter ***JP*** (mm)	1	1.57	1.85	1.91	1.88
volume removed V (mm^3^)	26.32	41.65	49.83	47.62	47.84
medium impact angle ***α**_**m**_* (°)	18	27	32	35	37
range of impact angles (°)	0–26	20–33	25.5–38	28–41	29–43.5

**Table 11:** Roughness of the specimen for a certain traverse speed and the rotational frequency.

Traverse Speed (mm/min)/Rotational Frequency (rpm)	Ra (μm)	Rz (μm)
10/250	5.65	38.302
20/250	6.039	37.541
40/250	9.705	57.78
40/500	8.561	55.534
40/1000	8.927	55.967
50/250	21.331	95.425
50/500	18.328	91.149
50/1000	22.521	97.601

**Table 12:** Roughness of the specimen for certain cumulative lateral jet shifts.

Cumulative Lateral Jet Shift (mm)	Ra (μm)	Rz (μm)
	lateral jet shift 0.75 mm	
1.5	6.9	8.79
3	8.037	10.308
4.5	8.537	10.722
	lateral jet shift 1 mm	
1	8.241	10.403
3	10.787	13.473
5	9.91	12.124

## Data Availability

No publicly archived datasets were reported or used.

## Nomenclature

***α_f_***	final impact angle on the edge of jet (°)
***α_i_***	initial impact angle on the edge of jet (°)
***α_m_***	angle of impact in the middle of impact zone (°)
***σ_m_***	tensile strength (MPa)
***σ_y_***	yield strength (MPa)
AWJT	abrasive waterjet turning
***D***	diameter of the jet (mm)
***E***	Young’s modulus (GPa)
***f***	frequency of rotation (rpm)
***H_B_***	Brinell hardness scale
***JP***	lateral jet position inside specimen diameter (mm)
***N***	number of passes
***v_p_***	traverse speed (mm/min)
***N_s_***	number of lateral jet shifts
***p***	pressure inside pumping system (MPa)
***R***	radius of the specimen (mm)
***R_a_***	average roughness of profile (μm)
***R_i_***	radius of the specimen for certain lateral jet shift (mm)
***R_z_***	mean peak to valley height of roughness profile (μm)
***s***	lateral jet shift (mm)
***s_c_***	cumulative lateral jet shift (mm)
***V***	volume of material removed during one lateral jet shift (mm^3^)
